# Delayed Post-Laparoscopic Sleeve Gastrectomy Leak Successfully Treated With Endoscopic Clips and Tissue Adhesive: Case Report and Literature Review

**DOI:** 10.7759/cureus.14532

**Published:** 2021-04-17

**Authors:** Abbas A Mohamed, Ahmed A Humaida, Ahmad S Qureshi

**Affiliations:** 1 Department of General and Laparoscopic Surgery, National Guard Hospital, Al Madinah, SAU; 2 Department of Internal Medicine, National Guard Hospital, Al Madinah, SAU

**Keywords:** laparoscopic sleeve gastrectomy, delayed leak, endoscopic chips, gastric leak, gi

## Abstract

Since it was first introduced, laparoscopic sleeve gastrectomy (LSG) has gained wide popularity and it is one of the most performed bariatric surgical procedures for weight reduction throughout the world. LSG is a simple and effective procedure for the reduction of excess body weight, but it is not without serious complications. We present a case of a 46-year-old obese male with multiple co-morbidities who presented with a delayed post-LSG leak that was successfully managed with endoscopic clips and tissue adhesive.

## Introduction

LSG has become one of the most frequently performed bariatric surgical procedures for obesity management throughout the world [1] Laparoscopic sleeve gastrectomy (LSG) is simple and efficient, and has a low rate of complication compared with the gastric bypass procedure. The post-laparoscopic gastric sleeve leak is the most feared complication of the procedure as it is associated with significant morbidity and mortality. We presented a case of a 46-year-old obese male with an estimated body mass index (BMI) of 46.2 kg/m^2^ who had a gastric leak three months after LSG. He presented with abdominal pain, fever, and vomiting for 48 hours. A CT scan of the abdomen showed leakage from the gastric remnant at the gastroesophageal junction and a small subdiaphragmatic and left perigastric collection. An upper gastrointestinal (GI) endoscopy confirmed the CT scan findings and provided successful management of both the abscess and the perforation.

## Case presentation

**Figure 1:**
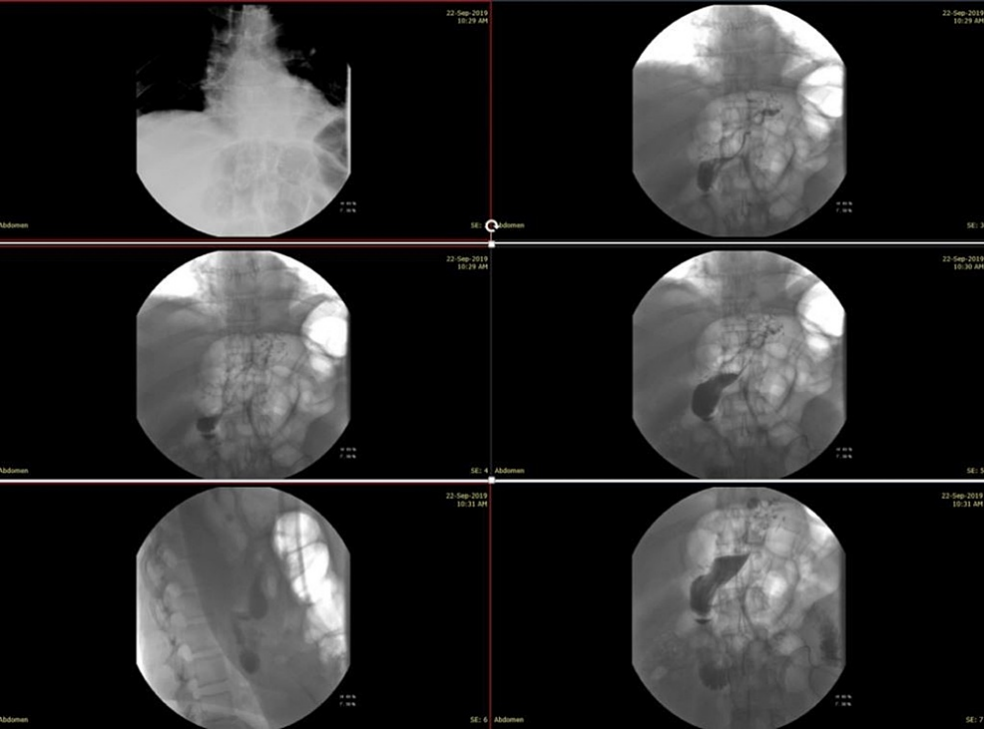
An oral contrast study with gastrografin showing the normal passage of contrast through the esophagus, the stomach to the small bowel without evidence of stenosis or leakage.

A 46-year-old morbidly obese man with an estimated BMI of 46.2 kg/m^2 ^was admitted for an LSG. He had a medical history of well-controlled hypertension and recurrent pulmonary emboli, and he was on the oral anticoagulant apixaban 2.5 mg daily. The patient’s preoperative investigations were all within the normal value, and his international normalized ratio (INR) was within the therapeutic range. Prior to surgery, he was started on deep vein thrombosis (DVT) and antibiotic prophylaxis (enoxaparin 40 mg subcutaneously daily for two weeks and cefazolin 2 grams intravenously once) according to the local hospital protocol. The operation was straightforward, with no intraoperative complications. The surgical technique involved devascularization of the greater curvature of the stomach by separating the greater omentum from the stomach using the LigaSure™ device (Medtronic, Minneapolis, MN, USA). Dissection was started 6 cm from the pylorus and proceeded upwards to the gastroesophageal junction. The gastric tube was calibrated with a 38-Fr calibration boogie and was stapled by Endo GIA™ reinforced stapler (Covidien, Minneapolis, MN, USA), without reinforcement sutures to the staple line. Towards the end of the procedure, the operative field was well inspected for bleeding and intra-operative leakage. An oral contrast study on the second postoperative day did not show any evidence of leakage or stenosis (Figure 1).

The patient was begun on oral fluid during the second postoperative day and was discharged on the third postoperative day. On outpatient follow-up, the patient was well with no complaints and had lost 15 kg within 30 days of surgery.

He presented three months later with symptoms of epigastric pain, vomiting, and fever for 48 hours. On examination, he was febrile with a temperature of 38.2°C without anemia or jaundice. Abdominal examination revealed mild tenderness over the left subcostal region. Examination of the other systems was normal. The laboratory investigations showed hemoglobin of 13.4 g/dL, hematocrit of 33.7%, and white blood cell count of 12.8 K/mm^3^ with 78% neutrophils. The PCR was 58 mg/L (normal value: 0.0-5 mg/L). Other blood tests, including urea and electrolytes, liver function tests, and coagulation profile were within normal limits.

**Figure 2:**
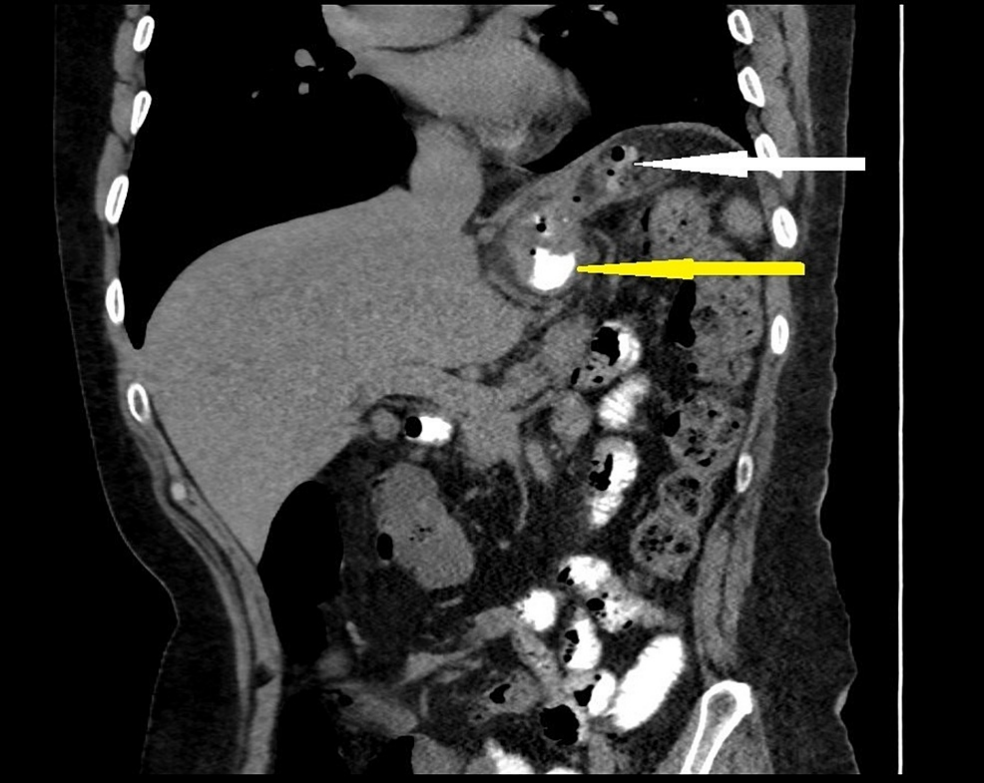
CT scan image (sagittal view) showing fluid collection next to the upper lateral aspect of the gastric tube with contrast leak (the white arrow). The yellow arrow illustrates the oral contrast within the gastric tube.

**Figure 3:**
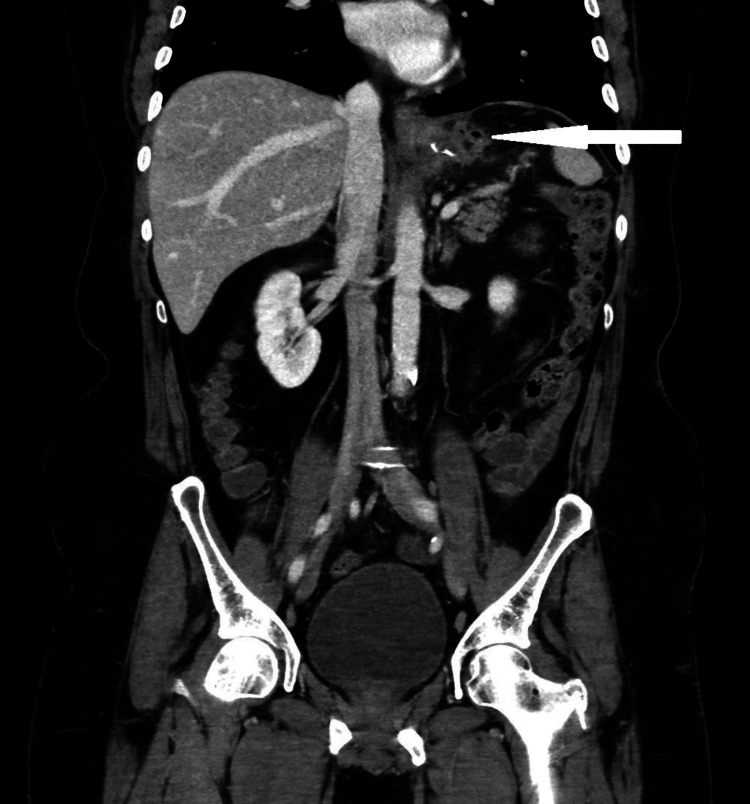
Abdominal CT scan image (coronal view) showing the fluid collection next to the upper lateral aspect of the gastric tube extending to the left subdiaphragmatic region with multiple air bubbles.

He underwent a double-contrast CT scan, which revealed circumferential mural thickening of the residual gastric tube with a left upper lateral gastric wall perforation of 1 to 1.5 cm just distal to the gastroesophageal junction. There was also a fluid collection next to the upper left lateral aspect of the gastric tube extending to the left subdiaphragmatic region, which measured 4 x 3 x 3 cm. Delayed oral contrast imaging revealed leakage of the contrast into the fluid collection (Figures 2, 3).

**Figure 4:**
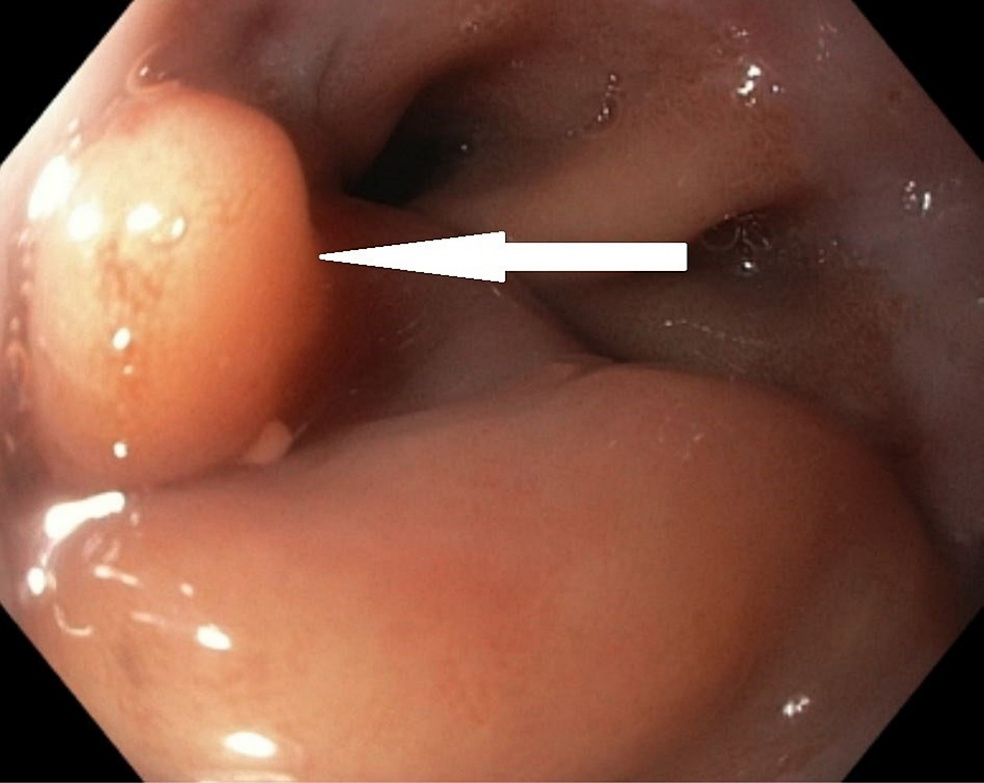
An endoscopic image showing the prominent mucosal fold or the flap overlying the perforation at the esophagogastric junction (the white arrow).

**Figure 5:**
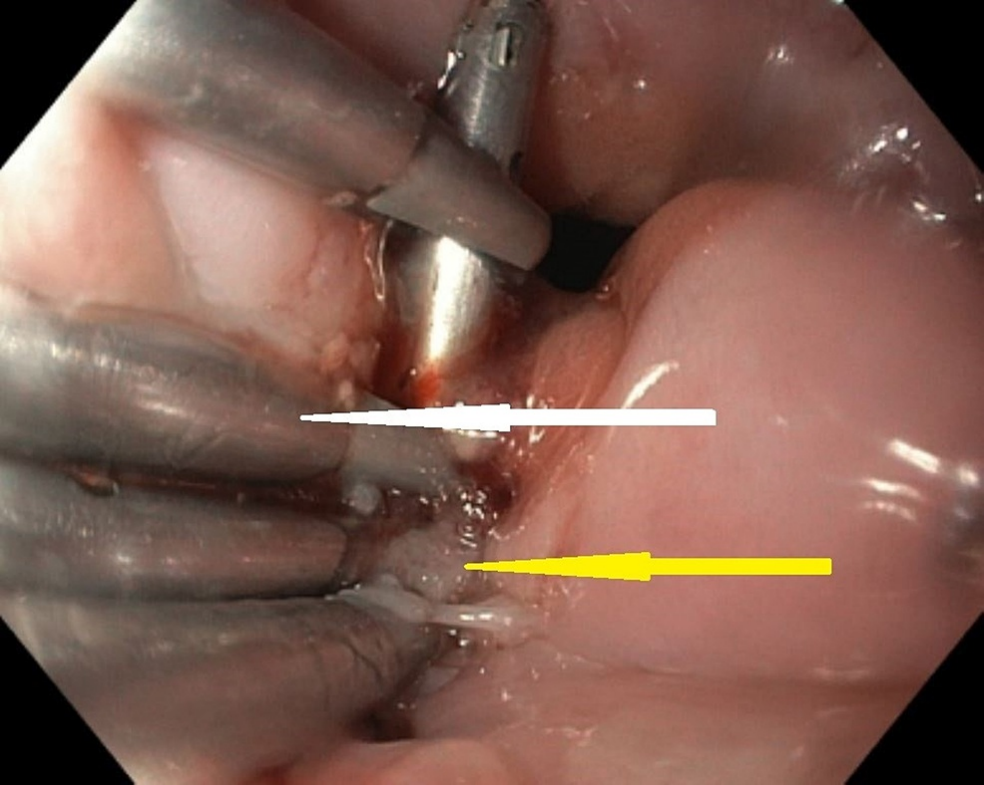
An endoscopic image showing the Resolution clips (the white arrow) and the tissue adhesive between the clips (the yellow arrow).

The patient underwent an upper GI endoscopy, which showed a mucosal fold or flap at the esophagogastric junction (Figure 4). On advancement of the scope between the fold and the wall, a purulent discharge pouring from underneath the fold was evident. Frequent flushing and suctioning identified a 1x1.5 cm perforation at the esophagogastric junction with the surgical staple at the distal end. Because of the unavailability of a proper stent, five Resolution™ clips (Boston Scientific, Marlborough, MA, USA) were applied to close the defect together with the injection of histoacryl (tissue adhesive comprises monomeric n-butyl-2-cyanoacrylate) between the clips (Figure 5). The endoscope was advanced through the gastric tube into the duodenum without hold, luminal narrowing, or stenosis.

 

**Figure 6:**
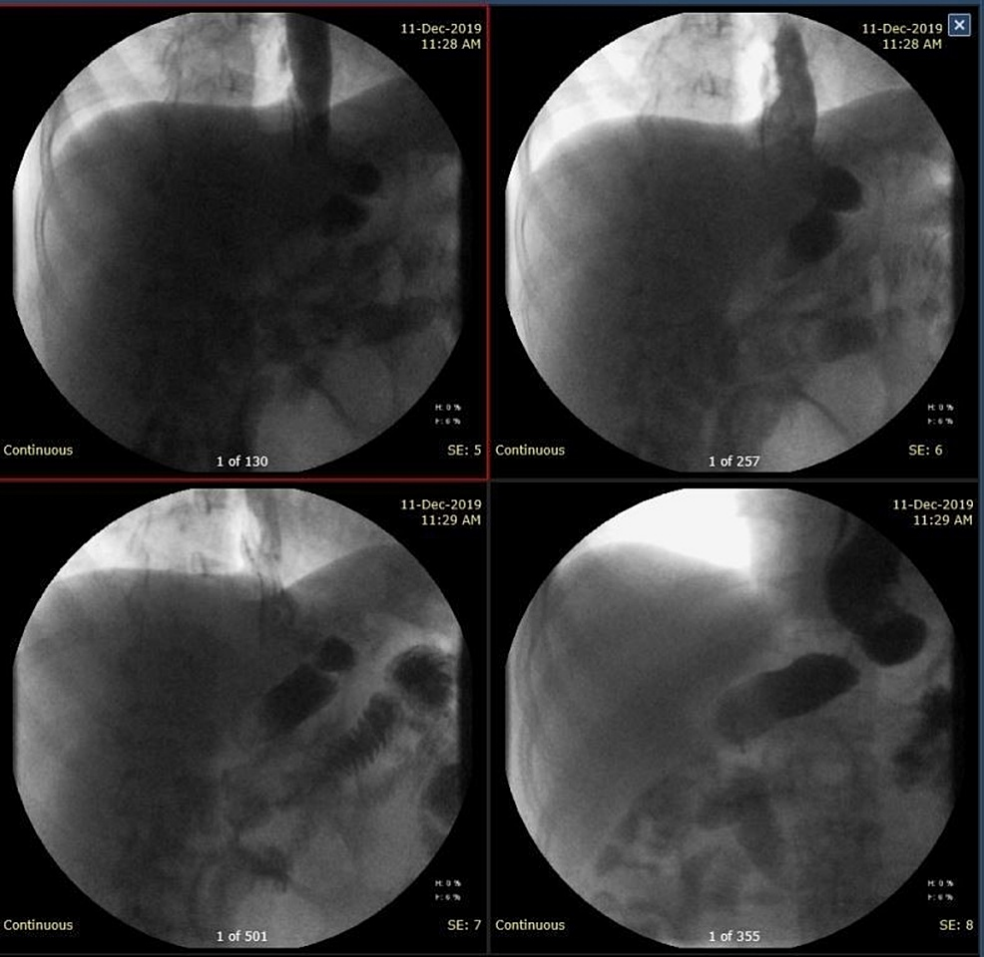
An oral contrast study with gastrografin after two months showing no leak from the gastric remnant.

**Figure 7:**
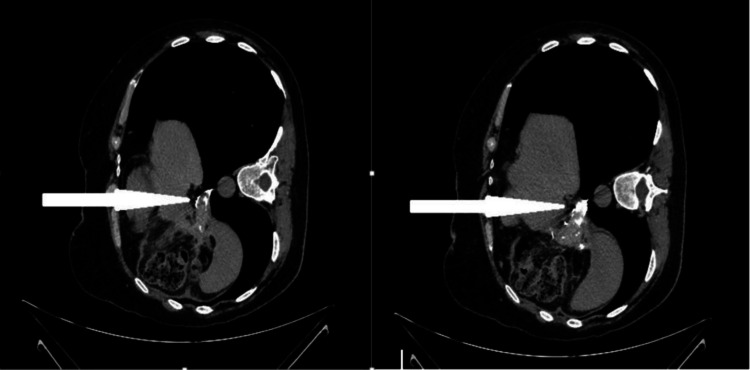
Abdominal CT scan images (axial views with the patient in left lateral position) showing complete resolution of the left subdiaphragmatic collection with no obvious contrast leak from the gastric tube (the white arrows).

After the procedure, the patient was started on oral fluid and slowly progressed to a solid diet over a month. He made an uneventful recovery. A gastrograffin study after two months did not show any leak on the first passage. A repeat CT scan of the abdomen demonstrated almost complete resolution of the left subdiaphragmatic collection with no obvious contrast leak from the gastric tube (Figures 6, 7).

## Discussion

Morbid obesity (BMI ≥ 40 kg/m^2^) has become a common epidemic in western cultures and is slowly involving the rest of the world [2]. According to the World Health Organization (WHO), in 2016, more than half of the world’s adult population had an elevated BMI, with 39% being overweight (BMI ≥ 25.0-29.9 kg/m^2^) and 13% being obese (BMI ≥ 30 kg/m^2^) [3]. The WHO expected that by 2030, more than 2.16 billion people will be overweight and 1.12 billion obese in the world [4].

Surgery is the most effective long-term treatment option for sustained weight loss and improvement in comorbidity in the morbidly obese [5]. The sleeve gastrectomy was first described by Hess and Hess in 1988 and subsequently popularized by Marceau et al. as a modification of biliopancreatic diversion that was first described by Scopinaro in 1997 [6,7]. With the progress of the minimally invasive techniques, Ren et al. performed the first laparoscopic LSG in 2000 as part of a duodenal switch procedure, and the role of the LSG continued to evolve [8,9].

Since it was first introduced, LSG gained wide popularity because of its simplicity, efficacy, and relatively low rate of complication. Currently, LSG has become the most frequently performed bariatric surgery procedure for the management of obesity throughout the world [1].

LSG reduces weight by combined anatomical effect attributed to the reduction of total gastric capacity, and a physiological effect attributed to the removal of fundal hunger hormone (ghrelin) producing cells [10].

Despite its popularity, LSG is not risk-free, with the most serious risk being the staple line leak. Staple line leak is the most feared complication of LSG as it is difficult to treat and is associated with significant morbidity and mortality. The local risk factors contributing to a leak are stapling devices’ mechanical failure, staple line dehiscence, infection, and ischemia due to poor blood flow which contributes to a decrease in oxygen and subsequent ischemia to the tissue [11,12].

Csendes et al. have described a classification system for gastric leaks based on three parameters: time of occurrence after surgery, severity, and location. The three categories are as follows: early leaks that occur one to four days after surgery, intermediate leaks that occur five to nine days after surgery, and late leaks that occur at day 10 or more after surgery [11].

The gastric leak tends to occur at the proximal third of the stomach, near the gastroesophageal junction, because of high pressure within the sleeve gastric tube, impaired peristaltic activity, and tissue ischemia [12,13]. Despite the fact that there are a large number of studies assessing various methods of making the staple line secure, there is to date no consensus on which technique is best for reducing the risk of a leak [14].

The clinical presentation of the early post-LSG leaks varies from mild symptoms to sepsis, septic shock, and multiple organ failure. The late presentation is usually in form of peri-sleeve abscesses and chronic fistulas [15].

Management of post-LSG leaks ranges from conservative management to aggressive surgical intervention depending on the time of occurrence after surgery, severity, and the anatomical location of the leak. Operative management is useful for debridement and drainage, but it often fails to close the defect in acute leaks due to inflammation and tissue friability [16]. Besides, radical surgery in the form of gastrectomy and oesophagojejunostomy is often associated with significant short-term postoperative complications and long-term nutritional deficiencies.

Endoscopic management of GI leaks provides a minimally invasive, safe, and efficient alternative for surgery in selected cases. It includes the use of fibrin glue, metallic stents, plugs, and clips [17].

Rogalski et al. [18] conducted a systematic literature search of the Medline/Scopus databases to identify full-text articles published up to February 2019 on the use of self-expandable stents, clipping, or tissue sealants as primary endoscopic strategies used for leak/fistula closure. They found that the success rate of self-expanding stents in the treatment of leaks/fistulas after bariatric surgery was 92%, with a 23% risk of stent migration. The success rate of the over-the-scope clips (OTSC) system was 67.1%, with a few complications (migration, stenosis, tear). Fibrin glue alone was used only in 10 patients with a 92.8-100% success rate of fistula closure that usually required repeated sessions. Minor complications of fibrin glue applications in the form of pain and fever occurred in 12.5% of patients. They concluded that endoscopic techniques are effective for the management of post-bariatric leaks and fistulas in properly selected patients.

Endoscopic clips (endoclips) have made a tremendous advance since the first description of their use in GI endoscopy by Hayashi et al. in Japan more than 35 years ago [19]. These advances revolutionized the endoscopic management of many GI emergencies. Endoclips were initially used for hemostasis of GI bleeding. However, their indications have expanded significantly to include the closure of GI leaks, fistulae, and perforations. They are being increasingly used in bariatric endoscopy for the primary management of LSG leaks with an overall success rate of 73-90% [20,21].

The selection of endoscopic methods of the treatment of post-LSG leaks depends on many factors including patient presentation, site and size of perforation, and available resources and expertise. In our case, we opted to use the endoclips because of the lack of a proper stent at the time.

## Conclusions

LSG has become the most frequently performed bariatric surgery procedure for the management of obesity throughout the world. Leak after sleeve gastrectomy is associated with significant morbidity and mortality and is one of the most feared complications of the procedure. As the clinical presentation of post-LSG leaks varies widely, as well as available methods of management, we recommend that every patient be assessed and managed individually. We also recommend a multidisciplinary approach to patients with post-LSG leak by a multiple medical specialty team including surgeons, interventional radiologist endoscopes, intensivists, and dietitians.
